# Nutritional Models of Experimentally-Induced Subacute Ruminal Acidosis (SARA) Differ in Their Impact on Rumen and Hindgut Bacterial Communities in Dairy Cows

**DOI:** 10.3389/fmicb.2016.02128

**Published:** 2017-01-25

**Authors:** Jan C. Plaizier, Shucong Li, Hein M. Tun, Ehsan Khafipour

**Affiliations:** ^1^Department of Animal Science, University of ManitobaWinnipeg, MB, Canada; ^2^Department of Medical Microbiology, University of ManitobaWinnipeg, MB, Canada

**Keywords:** subacute ruminal acidosis, rumen, cecum, bacteria, 16S rRNA gene sequencing, real-time quantitative PCR

## Abstract

Effects of subacute ruminal acidosis (SARA) challenges on the bacteria in rumen fluid, cecal digesta, and feces of dairy cows were determined using 16S rRNA gene pyrosequencing and real-time quantitative PCR. Six non-lactating Holstein cows with cannulas in the rumen and cecum were used in a 3 × 3 Latin square arrangement of treatments. During the first 3 wk of each experimental period, cows received a control diet containing 70% forages on a dry matter (DM) basis. In wk 4 of each period, cows received one of three diets: (1) the control diet; (2) a diet in which 34% of the dietary DM was replaced with pellets of ground wheat and barley (GBSC); or (3) a diet in which 37% of dietary DM was replaced with pellets of ground alfalfa (APSC). Rumen fluid, cecal digesta and feces were collected on d 5 of wk 4 of each period and the composition of the bacterial community was studied. Rumen fermentation responses were reported in a companion study. Both SARA-inducing challenges resulted in similar digesta pH depressions (as shown by the companion study), and reduced bacterial richness and diversity in rumen fluid, but GBSC had the larger effect. None of the challenges affected these measures in cecal digesta, and only GBSC reduced bacterial richness and diversity in feces. Only GBSC reduced the abundance of Bacteroidetes in rumen fluid. Abundances of limited number of bacterial genera identified by 16S rRNA gene sequencing in the rumen, cecum and feces were affected by the GBSC. The APSC did not affect any of these abundances. Both challenges increased the abundances of several starch, pectin, xylan, dextrin, lactate, succinate, and sugar fermenting bacterial species in the rumen, cecum, and feces as determined by qPCR. Only GBSC increased that of *Megasphaera elsdenii* in the rumen. Both challenges decreased the abundance of *Streptococcus bovis*, and increased that of *Escherichia coli*, in cecal digesta and feces, with GBSC having the larger effect. These results showed that the SARA challenges caused moderate and reversible changes of the composition of the bacteria in the foregut and hindgut, with the greater changes observed during GBSC.

## Introduction

In order to meet their production potential, high yielding dairy cows require high-energy diets. These diets commonly contain high inclusion rates of grains. These high inclusion rates affect conditions for microorganisms in the rumen and large intestine, including the acidity, osmolality, and the contents of fermentable substrates (Tajima et al., 2001; Plaizier et al., 2008, 2012). Increases of the dietary grain content and, as a result, the dietary starch contents can affect the rumen and hindgut bacteria, but these effects vary greatly among animals (Khafipour et al., 2009c; Mao et al., 2013; Petri et al., 2013). Feeding high-grain diets to cows creates the risk of subacute ruminal acidosis (SARA), a metabolic disorder characterized by a reversible rumen pH depression for extended periods each day (Plaizier et al., 2008; Kleen and Cannizzo, 2012). Also, experimentally induced SARA by feeding high-grain diets increases concentrations of free bacterial lipopolysaccharide endotoxin (LPS) both in the rumen and hindgut digesta, and triggers an immune response in dairy cows (Li S. et al., 2012; Plaizier et al., 2012). The induction of SARA also reduces bacterial richness and diversity in the rumen and leads to a decline in Bacteroidetes and an increase in Firmicutes abundance in the rumen (Khafipour et al., 2009b,c; Mao et al., 2013; Petri et al., 2013). Grain-induced SARA can also affect the bacteria and increase fermentation in the hindgut, most likely via increasing by-pass starch that escapes rumen fermentation and small intestine digestion, which increases acidity and volatile fatty acid (VFA) concentrations of digesta in the hindgut and the feces (Khafipour et al., 2009c; Mao et al., 2012; Petri et al., 2013). Feeding diets that contain ground forages, such as pellets of ground alfalfa hay, can induce SARA without increasing the starch contents of digesta in the hindgut and feces (Khafipour et al., 2009b; Plaizier et al., 2012). Also, Khafipour et al. (2009b) and Li S. et al. (2012) observed that replacing alfalfa hay with pellets made of ground alfalfa hay resulted in a rumen pH depression representative of SARA, without causing the innate immune response that occurs during grain-induced SARA (Plaizier et al., 2008, 2012). These differences between the two models of SARA induction may be the result of differences in the impact of these challenges on the bacteria in the rumen and the hindgut (Khafipour et al., 2009c; Plaizier et al., 2012). This may be expected, as the composition of digesta and the conditions and availability of substrates for the bacteria in the digestive tract vary between these two SARA-induction models (Khafipour et al., 2009a,b). It has been hypothesized that the differences between the two-SARA induction models may be caused by the increase in rumen by-pass starch during grain-induced SARA that results in an increase in the lysis and shedding of LPS by gram-negative bacteria in the hindgut that triggers the immune response (Khafipour et al., 2009a,b; Plaizier et al., 2012). These hypotheses challenge the current definition of SARA, which is only based on a rumen pH depression.

Several studies on the impact of grain-induced rumen acidosis on rumen bacteria of dairy cows have been conducted using culture-based, quantitative PCR (qPCR), and fragmentation techniques, such as terminal restriction fragments length polymorphism (Nagaraja et al., 1978; Russell and Hino, 1985; Khafipour et al., 2009c). Recent advances in sequencing technology, however, offer rapid, low-cost molecular-based methodologies that can investigate bacterial communities with high resolution and as a whole (Krause et al., 2013). Using these techniques, Mao et al. (2013) and Li et al. (2013) reported that grain-induced SARA increased the abundance of Firmicutes and decreased that of Bacteroidetes in the rumen. However, there were discrepancies in the proportion of lower-abundance phyla, such as Proteobacteria, Actinobacteria, Spirochaetes, and Tenericutes and the shift in their proportions due to induction of SARA. These authors also investigated the impact of grain-induced SARA on the fecal bacteria of dairy cows and reported associations among the abundances of several species of Bacteroidetes and Firmicutes and the concentrations of VFA in the feces, which suggests that such associations exist in hindgut digesta also. The conflicting and inconsistent results among studies may be caused by the complexity of the bovine gut microbiota, the difference between experimental approaches and sequencing techniques, the small scale of these studies, and the variation among cows in the susceptibility to SARA. Studies that compared the effects of different models of SARA induction on the bacteria of the rumen and the large intestine within the same experiment have not yet been conducted. Such studies are needed to enhance the understanding of the relationship between the SARA and the gut bacteria of dairy cows, as this will lay the foundation for the development of strategies to prevent this disorder.

This report is part of a larger experiment in which both a grain and a finely ground alfalfa hay SARA challenges were induced in dairy cows. The companion study of Li S. et al. (2012) described the effects of those challenges on the pH and the concentrations of VFA and free LPS of digesta in the foregut and hindgut. The current report describes the effects of this challenge on the microbiota in these digesta that occurred in the same experiment.

We hypothesized that SARA induced by high-grain feeding and SARA induced by feeding pellets of ground forage alter the bacteria of rumen digesta, cecum digesta and feces, but that these effects differ between the two models. In this study, we used pyrosequencing technology and qPCR to investigate and compare the impact of these two models on the bacteria of digesta in the rumen and the cecum, and in the feces of non-lactating dairy cows.

## Materials and methods

### Animals models and experimental treatments

The design of the study was described earlier in the companion manuscript of Li S. et al. (2012) that described effects of the experimental treatments on fermentation and endotoxins in the rumen and the hindgut. The study was pre-approved by the Fort Garry Campus Animal Care Committee of the University of Manitoba in accordance with the Canadian Council for Animal Care guidelines (Canadian Council on Animal Care (CCAC), 2009). In brief, six non-lactating, multiparous Holstein cows with cannulas in the rumen and cecum were used. The animals had live weights of 620 ± 45.7 kg (mean ± SD). They were randomly allocated to three treatments in a 3 × 3 Latin square design experiment which consisted of three periods of 4 weeks.

In the first 3 weeks of each experimental period, all cows received a basal diet with a forage-to-concentrate ratio of 70:30. Starting the Friday of the 3rd week to Monday of the 4th week of each experimental period, the diets of three groups of cows were changed as follows: (1) the basal diet remained unchanged (control), (2) the grain pellets consisting of 50% ground wheat and 50% ground barley gradually replaced with 34% of the dry matter of the basal diet (Grain-based SARA challenge, GBSC); and (3) alfalfa pellets were added up to 37% of the basal diet DM to replace alfalfa hay (alfalfa-pellet SARA challenge, APSC). Three diets were then fed to all cows in the remainder of 4th week. Experimental diets were described in detail by Li S. et al. (2012). A summary of the chemical composition of these diets is given in Table 1.

**Table 1:** **Chemical composition of experimental diets**.

**Item**	**Nutrient composition**		
	**Control**	**APSC**	**GBSC**
Dry matter, %	54.3	69.0	61.6
Crude protein, % DM	16.1	16.0	16.0
Neutral detergent fiber, % DM	35.6	34.5	22.9
Starch, % DM	14.5	15.9	33.7

*Control, Control; APSC, alfalfa-pellet SARA diet; GBSC, grain-based SARA challenge diet*.

Cows were housed in individual stalls in the large animal metabolism facility of the Glenlea Research Station, University of Manitoba, and were cared for in accordance with the Canadian Council for Animal Care guidelines (Canadian Council on Animal Care (CCAC), 2009). Cows were fed *ad libitum* once daily at 0900 h, allowing for between 5 and 10% of feed refusals, and had free access to fresh water.

### Rumen fluid and cecum digesta sampling

Rumen fluid, digesta in the cecum, and feces were sampled on d5 of wk 4 of each experimental period at 6 h after feed delivery. Rumen fluid was collected from the ventral sac of the rumen and strained through 4 layers of sterile cheesecloth. Cecal digesta was collected via the cecal cannula and fecal samples were collected from the rectum. All samples were then aliquoted into 10 ml sterile tubes or Whirl-Pak 60 g bags (NASCO, WI, USA) before they were snap frozen in liquid nitrogen and stored in −80°C until further analysis.

### DNA extraction

Rumen fluid and digesta samples were thawed at room temperature and subsequently kept on ice. A total of 1 ml of rumen fluid was centrifuged at 15,000 × g to collect the sediment. Subsequently, DNA was extracted from the sediment using a ZR fecal DNA kit (D6010; Zymo Research Corp., Orange, CA, USA) that included a bead-beating step for disrupting bacterial cells. The extraction of DNA of cecal and fecal samples was conducted on 200 mg of sample using the same kit. At the last step of the procedure, DNA was eluted from the column with elution buffer, and DNA was quantified using a NanoDrop 2000 spectrophotometer (Thermo Scientific, Waltham, MA, USA). The DNA samples were normalized to 20 ng/μl for pyrosequencing and to 2 ng/μl for qPCR. All DNA were quality verified by PCR amplification of the 16S rRNA gene using universal primers 27F (5′-GAAGAGTTTGATCATGGCTCAG-3′) and 342R (5′-CTGCTGCCTCCCGTAG-3′) as described (Khafipour et al., 2009c). Amplicons were verified by agarose gel electrophoresis. For qPCR analyses, DNA samples were aliquoted into 10 μl/vials, which was sufficient for testing one set of primers, in order to avoid repeated freeze-thaw cycles. All DNA samples were stored at −80°C.

### 16S rRNA gene sequencing and bioinformatics

A total of 54 DNA samples from rumen fluid samples were pyrosequenced using the bacterial tag-encoded GS FLX-Titanium amplicon as described by Dowd et al. (2008b). In brief, a mixture of Hot Start, HotStar high fidelity Taq polymerases, and Titanium reagents were used to perform a one-step PCR (35 cycles) with primer 28f (5′-GAGTTTGATCNTGGCTCAG-3′) and 519r (5′-GTNTTACNGCGGCKGCTG-3′), which covered the hypervariable regions V1-V3 of the bacterial 16S rRNA genes (Dowd et al., 2008a). The pyrosequencing procedures were carried out at the Research and Testing Laboratory (Lubbock, TX; http://www.Researchandtesting.com). The raw data are presented in Supplementary Data Sheet 1.

### Sequence editing, classification, and building of the phylogenetic tree

Pyrosequencing data were binned using sample-specific barcode sequences, and filtered using QIIME 1.7 (Caporaso et al., 2010b). All sequences <200 bp, with ambiguous nucleotide bases, or a homopolymer length longer than 7 bp were removed from downstream analyses. Chimeric sequences were detected using the UCHIME algorithm (USEARCH 6.1) and sequences were assigned to Operational Taxonomic Units (OTU) using the QIIME implementation of UCLUST (Edgar et al., 2011). In total, 71,029 sequences (6733 unique observations) from 18 ruminal samples, 113,734 sequences (10,363 unique observations) from 18 cecal samples and 104,277 sequences (10,784 unique observations) from 18 fecal samples were generated in this step. An open reference-based OTU picking approach was implemented with the QIIME algorithm and usearch61 method with default parameters (Edgar et al., 2011) were used to cluster the sequences at the 97% sequence similarity level using the Greengene database (Version 13_5) (McDonald et al., 2012). Those sequences that failed to cluster were subsampled for de novo OTU picking. All picked OTUs were subsequently aligned by PyNAST (Caporaso et al., 2010a), and a phylogenetic tree was built using FastTree method (Price et al., 2009) to calculate UniFrac distances (Lozupone et al., 2011) within QIIME. Taxonomy was assigned to OTUs using RDP classifier via QIIME with a confidence threshold of 0.8 (Wang et al., 2007).

### Alpha- and beta-diversity analyses

**Figure 1:**
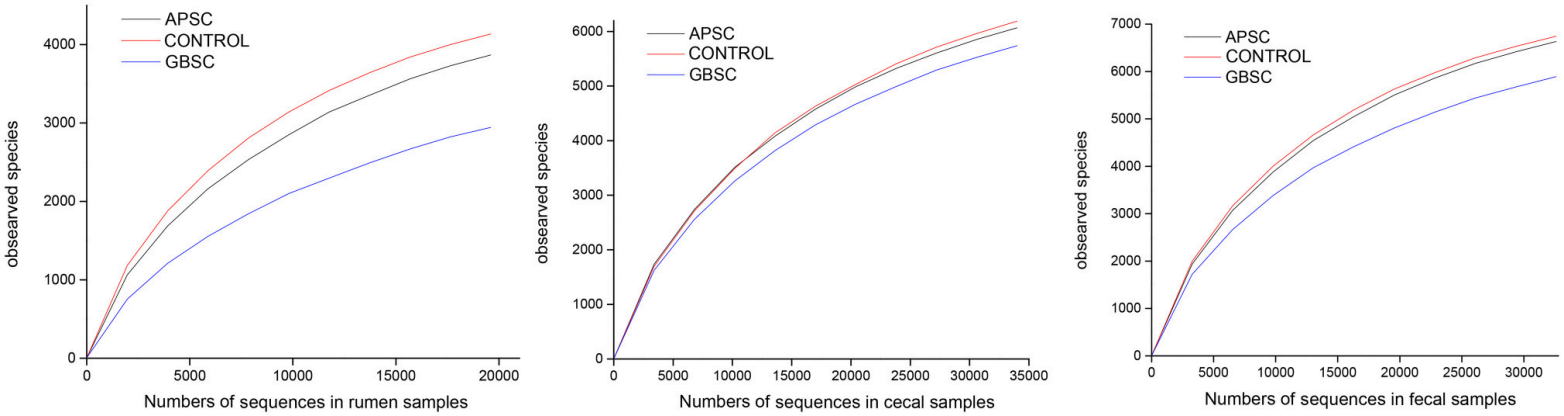
**Rarefaction curves indicating the observed number of operational taxonomic units (OTUs) at a genetic distance of 3% in rumen bacterial communities of dairy cows under control feeding, an alfalfa-pellet SARA challenge (APSC) or a grain-based SARA challenge (GBSC) conditions**. The number V1–V3 sequences of 16S rRNA gene in the pyrosequencing library was the pooled reads across individual samples (6 samples).

Samples were rarefied for alpha-diversity calculations and generation of rarefaction curves (Figure 1), in order to eliminate the bias caused by the different sample sizes (Roesch et al., 2007). Standard alpha-diversity indices were determined. The α parameter of Fisher's log-series was used as a diversity index (Fisher et al., 1943). Richness indices included the Chao1 index and the abundance based coverage estimation (ACE) richness indices. Diversity estimators included the Shannon and Simpson indices (Hill, 1973).

**Figure 2:**
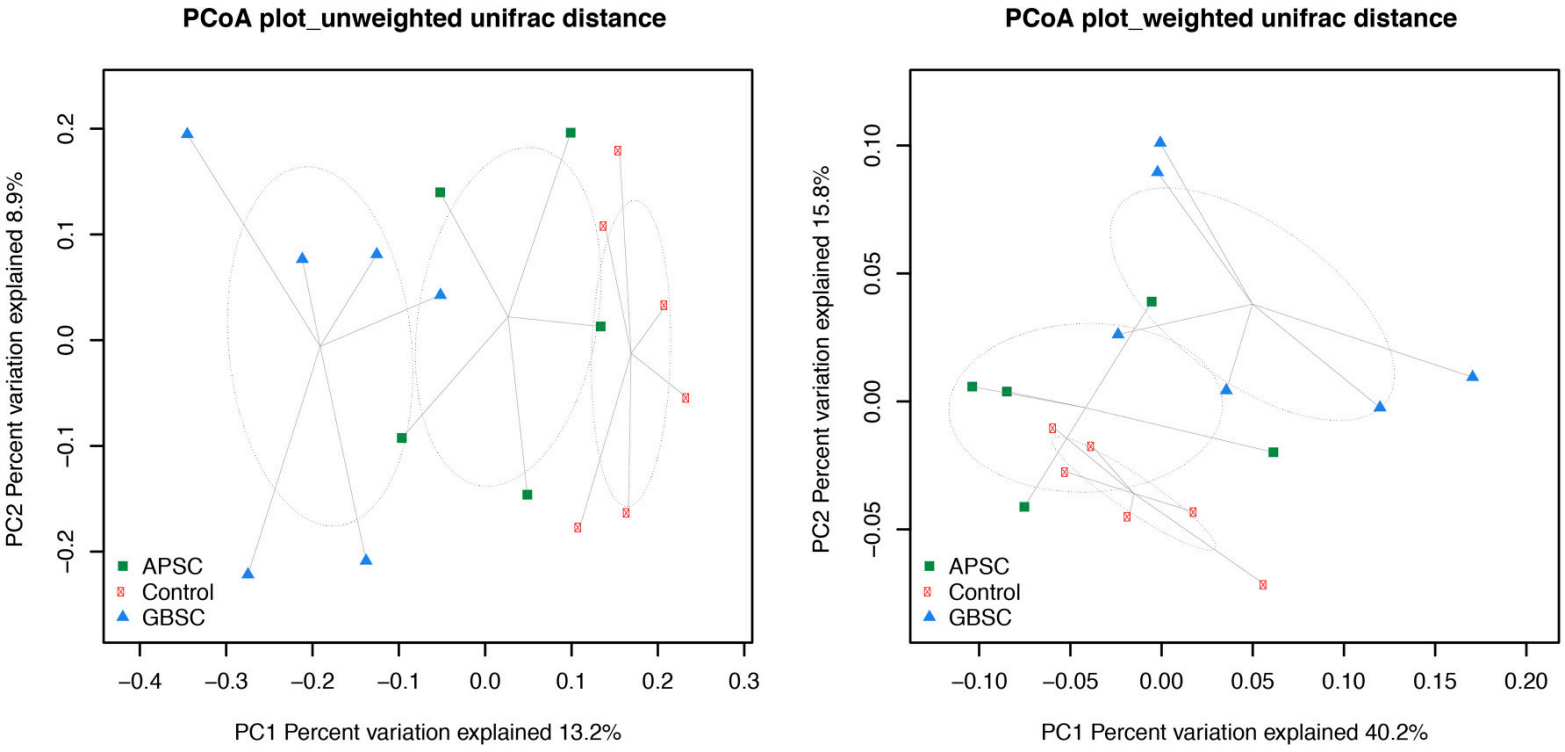
**Two-dimensional PCoA plots based on the unweighted and weighted UniFrac distance matrix illustrates variation in rumen fluid bacterial communities as affected by different subacute ruminal acidosis (SARA) challenge conditions**. The ellipses were drawn with standard errors of the points at 0.95 confidence limit. Labels are placed at means centers of each site and are linked to each sample of the corresponding site. Abbreviations in figure: APSC, alfalfa-pellet SARA challenge; GBSC, grain-based SARA challenge. Significance levels unweighted analysis, APSC vs. Control *P* = 0.01; GBSC vs. Control *P* < 0.01; GBSC vs. APSC *P* = 0.15. Significance levels weighted analysis, APSC vs. Control *P* = 0.22; GBSC vs. Control *P* < 0.01; GBSC vs. APSC *P* = 0.06.

**Figure 3:**
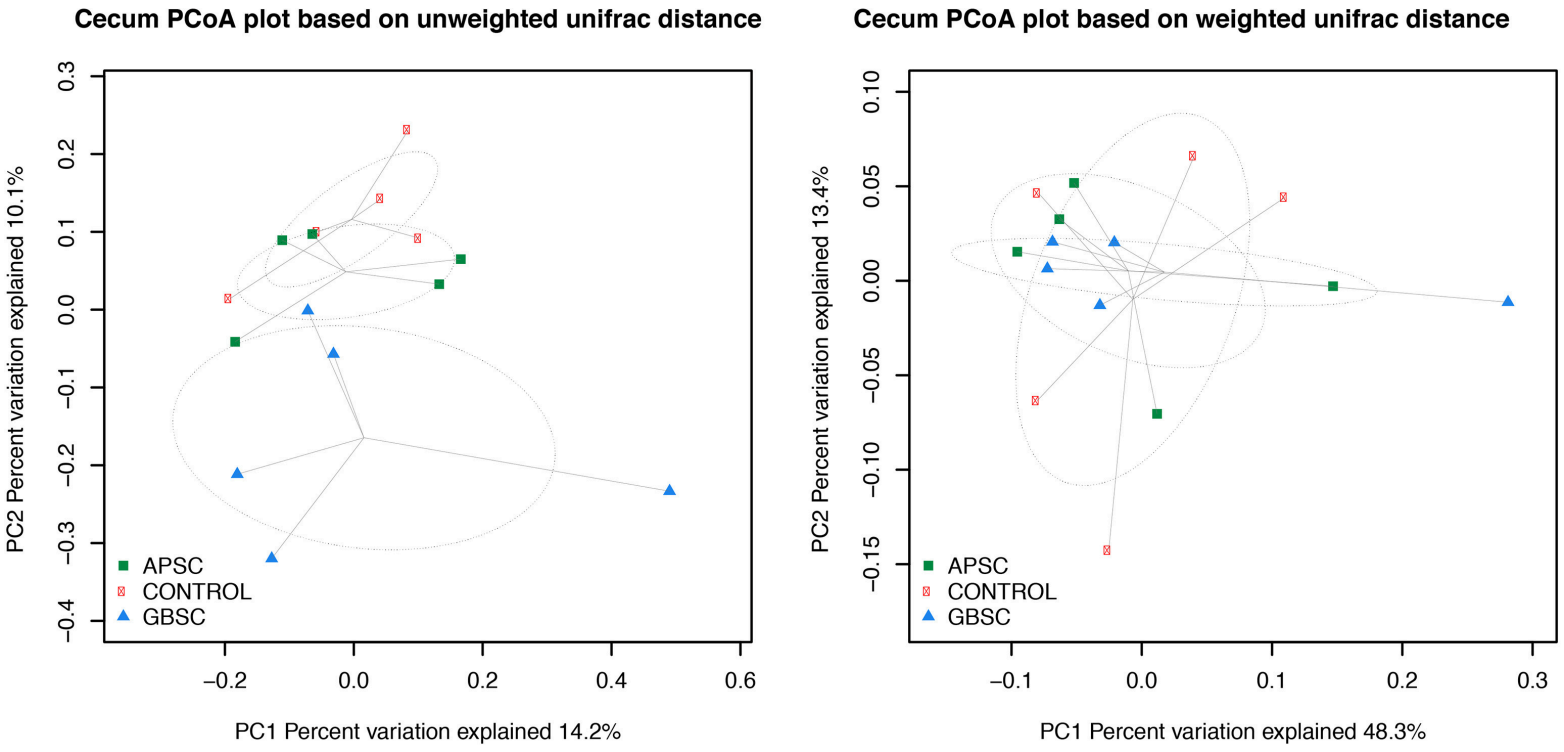
**Two-dimensional PCoA plots based on the unweighted and weighted UniFrac distance matrix illustrates variation in cecal bacterial communities as affected by different subacute ruminal acidosis (SARA) challenge conditions**. The ellipses were drawn with standard errors of the points at 0.95 confidence limit. Labels are placed at means centers of each site and are linked to each sample of the corresponding site. Abbreviations in figure: APSC, alfalfa-pellet SARA challenge; GBSC, grain-based SARA challenge. Significance levels unweighted analysis, APSC vs. Control *P* = 0.53; GBSC vs. Control *P* = 0.05; GBSC vs. APSC *P* = 0.26. Significance levels weighted analysis, APSC vs. Control *P* = 0.75; GBSC vs. Control *P* = 0.56; GBSC vs. APSC *P* = 0.77.

**Figure 4:**
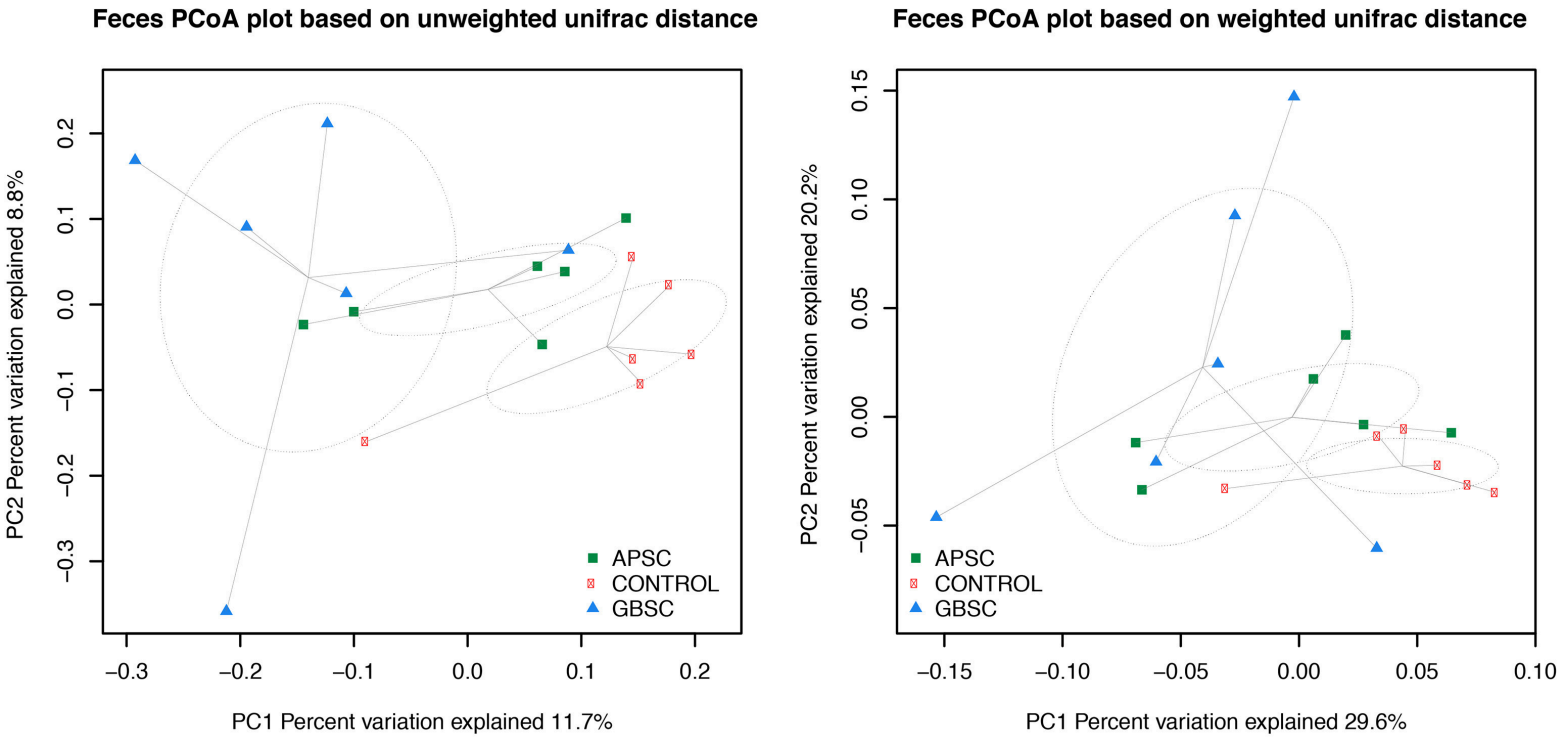
**Two-dimensional PCoA plots based on the unweighted and weighted UniFrac distance matrix illustrates variation in fecal bacterial communities as affected by different subacute ruminal acidosis (SARA) challenge conditions**. The ellipses were drawn with standard errors of the points at 0.95 confidence limit. Labels are placed at means centers of each site and are linked to each sample of the corresponding site. Abbreviations in figure: APSC, alfalfa-pellet SARA challenge; GBSC, grain-based SARA challenge. Significance levels unweighted analysis, APSC vs. Control *P* = 0.01; GBSC vs. Control *P* < 0.01; GBSC vs. APSC *P* = 0.15. Significance levels weighted analysis, APSC vs. Control *P* = 0.05; GBSC vs. Control *P* < 0.01; GBSC vs. APSC *P* = 0.28.

The dataset was also subsampled to the median (de Cárcer et al., 2011) for beta-diversity analysis using Phyloseq (McMurdie and Holmes, 2013). UniFrac-based principal coordinates analysis (PCoA; Figures 2–4) were conducted with Phyloseq. PCoA plots were generated based on both weighted and unweighted UniFrac distance matrix. In addition, permutational multivariate analysis of variance (PERMANOVA; Anderson et al., 2011) based on the same similarity matrix were used to test the effect of the treatments.

### Partial least square discriminant analyses

Partial least square discriminant analysis (PLS-DA; SIMCA P+ 13.0, Umetrics, Umea, Sweden) was performed on the proportional data at the genus level to test the effects of treatments (Li R. et al., 2012) (Supplementary Figures 1–3). The PLS-DA is a particular case of partial least square regression analysis in which Y is a set of binary (0 vs. 1) variables describing the categories of a categorical variable on X. In this case, X variables were bacterial genera and binary Y was observations of control, GBSC, and APSC. For this analysis, data were scaled using Unit Variance in SIMCA. Cross-validation then was performed to determine the number of significant PLS components and a permutation testing was conducted to validate the model. R^2^ estimate then was used to evaluate the goodness of fit and Q^2^ estimate was used to evaluate the predictive value of the model. The PLS-regression coefficients were used to identify genera that were most characteristic of each treatment group. The significant shifts of taxa were determined when the error bars of each component was above or below x axis of coefficient plot (Wang et al., 2016). The results of PLS-DA were visualized by PLS-DA loading scatter plots (Supplementary Figures 1–3).

### Quantitative PCR analysis

Quantitative PCR was carried out in 96-well optical plates on an AB 7300 system (Applied Biosystems, Foster City, CA, USA) as described previously (Khafipour et al., 2009c). The primers listed in Supplementary Table 1 were synthesized by University Core DNA Services (University of Calgary, Calgary, AB, Canada). Each reaction mixture was run in triplicate in a volume of 15 μl in optical reaction plates (Applied Biosystems, Foster City, CA, USA) sealed with optical adhesive film (Applied Biosystems, Foster City, CA, USA). Amplification reactions were carried out with 7.5 μl Power SYBR green PCR master mix (Applied Biosystems, Foster City, CA, USA) mixed with the selected primer set (Supplementary Table 1) with a final concentration of 450 nM. Amplification consisted of one cycle of 95°C (10 min) to activate AmpliTaq Gold polymerase, followed by 40 cycles of denaturation at 95°C (15 s), and annealing/extension at 60°C (1 min). Final melting analysis was obtained by slow heating from 65° to 95°C in order to assess the specificity of the prime set. The efficiency of the amplification of each primer set was calculated from the slope of the standard curve generated with pool DNA samples. The change in the quantity of target species in a tested samples relative to the same target species in a calibrator sample was calculated after all real-time data were normalized for Eubacteria using bacteria 16S RNA gene primer sets, which detect all bacterial strains (Khafipour et al., 2009c).

### Statistical analyses

The effects of treatment on alpha-diversity indices, bacterial abundances at the phylum and lower taxonomical levels, and species relative ratios were analyzed with SAS version 9.3 (SAS Institute Inc., 2011). The UNIVARIATE procedure was used to test the normality of error terms. Non-normally distributed data were transformed using the Box-Cox power transformation implemented within TRANSREG procedure that iteratively tests a variety of λ and identifies the best power transformation. Normalized data were used to assess the effect of treatment using MIXED procedure of SAS (SAS Institute Inc., 2011), with treatment and experimental period as fixed factors. The effect of cow was treated as random in the model. Pairwise comparisons between the treatments were conducted with Tukey's honestly significant difference test corrected for multiple comparisons. Statistical differences were declared as significant and highly significant at *P* < 0.05 and *P* < 0.01, respectively. Trends toward significance were discussed at 0.05 < *P* < 0.10.

## Results

### Bacterial richness and diversity

The rarefaction curves of the observed number of OTUs are given in Figure 1. Effects of the treatments on measures of bacterial richness and diversity in rumen fluid, cecal digesta and feces are reported in Table 2. Both SARA induction models reduced the bacterial richness and diversity in the rumen, but GBSC lead to the greatest reductions. The GBSC treatment reduced the richness of species, as indicated by the reduction of the numbers of OTUs classified at 97% distance of amplified 16S rRNA gene sequences and tended to reduce the effective number of species calculated from Simpson's reciprocal. Moreover, the GBSC treatment reduced the Fisher Richness Index and tended to reduce the Chao1 and ACE indices. In contrast, the APSC only tended to reduce the numbers of OTUs and the Fisher index. When both evenness and richness of the bacterial communities were considered, the GBSC treatment also had a greater impact than the APSC treatment, as indicated by the lower Shannon index of the GBSC. In the cecum, both SARA challenges did not affect the measures of the bacterial richness and diversity. The GBSC treatment reduced the Fisher and Shannon indices, and tended to reduce the number of OTUs and the effective number of species in feces. In contrast, APSC did not affect the richness and diversity in the feces.

**Table 2:** **Summary statistics of sequences in rumen fluid, cecum digesta and feces of dairy cows during Control, an alfalfa-pellet SARA challenge (APSC) or a grain-based SARA challenge (GBSC) treatment, including number of OTU^1^ (97% distance), Fisher index, richness indices (Chao1 and ACE), diversity indices (Shannon and Simpson), and effective number of species (Simpson's reciprocal)**.

	**Control**	**APSC**	**GBSC**	**SEM**	***P*****-value**
**RUMEN FLUID**					
Number of OTU^1^ (97% distance)	1031^a^^A^	714^a^^b^^B^	618^b^^C^	95	0.03
Fisher^2^	854^a^^A^	450^a^^b^^B^	338^b^^C^	107	0.02
Chao1	2540^A^	1514^AB^	1363^B^	345	0.06
ACE	2725^A^	1630^AB^	1579^B^	387	0.07
Shannon	6.41^a^	5.86^ab^	5.07^b^	0.28	0.03
Simpson	0.99	0.99	0.96	0.02	0.13
Simpson's reciprocal	302^A^	136^AB^	56^B^	73	0.09
**CECAL DIGESTA**					
Number of OTU^1^ (97% distance)	1973	1782	1679	97	0.17
Fisher^2^	1102	972	952	65	0.29
Chao1	3569	2948	3722	580	0.63
ACE	3728	3275	3739	498	0.77
Shannon	6.81	6.65	6.47	0.29	0.72
Simpson	1.00	0.99	0.99	0.01	0.83
Simpson's reciprocal	312	265	348	83	0.73
**FECES**					
Number of OTU^1^ (97% distance)	2188^A^	2056^A^	1773^B^	102	0.06
Fisher^2^	1375^a^	1237^ab^	908^b^	103	0.03
Chao1	3986	3213	3085	560	0.5
ACE	4104	3470	3132	408	0.28
Shannon	7.21^a^	7.11^ab^	6.77^b^	0.1	0.03
Simpson	1.00	1.00	1.00	0.01	0.21
Simpson's reciprocal	737^A^	560^AB^	392^B^	95	0.08

a,b *Treatments that do not share a letter had significantly different results by Tukey's Honest Significant Difference (HSD) test at a P < 0.05, corrected for multiple comparisons*.

A,B,C *Treatments that do not share a letter had significantly different results by Tukey's honestly significant difference (HSD) test at a P < 0.10, corrected for multiple comparisons*.

1 *OUT, operational taxonomic units*.

2 *The α parameter of Fisher's log-series was used as a diversity index (Fisher et al., 1943)*.

### Phylogenetic–based sample clustering

The results of PCoA analysis of the rumen, cecum, and feces data are given in Figures 2–4, respectively. Based on the weighted and unweighted UniFrac distances the control and GBSC treatments separated (*P* < 0.01) into distinct clusters in the rumen, whereas only the unweighted UniFrac distances showed that APSC clustered separately (*P* < 0.01) from the control treatment. The GBSC and APSC treatments only tended to cluster differently (*P* = 0.06) in the rumen when their weighted UniFrac distances were tested. In the cecum, only the GBSC and control treatment clustered separately based on their unweighted UniFrac distances (*P* = 0.05). Based on their unweighted UniFrac distances in feces, GBSC and APSC clustered differently (*P* < 0.01) from control. Based on the weighted UniFrac distances, a difference (*P* < 0.01) in clustering was observed between GBSC and control. The APSC and control clusters only tended (*P* = 0.05) to differ. In feces, GBSC and APSC only tended to cluster differently (*P* = 0.05) based on their unweighted UniFrac distances. Based on their weighted UniFrac distances, the GBSC and APSC treatments did not cluster differently in feces.

### Bacterial community composition

#### Phylum level

The vast majority (>99%) of the sequences of V1–V3 region of 16S rRNA gene were assigned to seven dominant phyla (abundance above 0.1% of the total community) in the rumen, cecum, and feces, including Actinobacteria, Bacteroidetes, Cyanobacteria, Firmicutes, Proteobacteria, Spirochaete, and Tenericutes (Table 3). Bacteroidetes and Firmicutes were most abundant phyla in all three compartments, and comprised about 90% of each community. The relative abundances of Bacteroidetes and Firmicutes were equal in the rumen, whereas Firmicutes dominated in the cecum and feces.

**Table 3:** **Relative abundance of phyla (above 0.1% of community) in rumen fluid, cecum digesta and feces of dairy cows fed a control diet or on cows given an alfalfa-pellet SARA challenge (APSC) or a grain-based SARA challenge (GBSC)**.

**Phyla in each compartment**	**Percentage of sequences in:**			**SEM**	***P*****-value**
	**Control**	**APSC**	**GBSC**		
**RUMEN**					
Bacteroidetes	48.9^a^	49.6^a^	41.9^b^	2.3	<0.01
Firmicutes	43.0	41.8	52.2	3.9	0.13
Spirochaetes	3.8	3.3	0.9	2.1	0.19
Tenericutes	1.1^a^	0.9^a^	0.4^b^	0.1	<0.01
Proteobacteria	0.56	0.73	0.30	0.21	0.16
Actinobacteria	0.37	0.26	3.24	1.96	0.58
Fibrobacteres	0.35	0.59	0.32	0.11	0.24
SR1	0.25^a^^A^	0.14^ab^^B^	0.02^b^^B^	0.05	0.02
Cyanobacteria	0.18^ab^^A^	0.32^a^^A^	0.01^b^^B^	0.08	0.01
TM7	0.10	0.06	0.14	0.06	0.29
**CECUM**					
Firmicutes	69.9	71.4	66.6	8.66	0.93
Bacteroidetes	22.7	21.5	25.8	5.36	0.85
Fusobacteria	3.8	2.8	4.9	3.96	0.35
Spirochaetes	0.56	1.22	1.03	0.45	0.74
Proteobacteria	0.35	0.48	0.23	0.12	0.31
Tenericutes	0.47	0.30	0.27	0.16	0.36
Lentisphaerae	0.42^a^	0.25^a^	0.05^b^	0.10	0.01
Verrucomicrobia	0.17^AB^	0.66^A^	0.07^B^	0.10	0.06
Actinobacteria	0.13	0.28	0.22	0.14	0.53
Cyanobacteria	0.12	0.20	0.03	0.05	0.29
Fibrobacteres	0.05	0.11	0.18	0.12	0.22
**FECES**					
Firmicutes	77.7	75.1	74.7	3.02	0.67
Bacteroidetes	18.4	20.8	21.0	2.63	0.65
Proteobacteria	0.48	0.38	0.50	0.13	0.83
Spirochaetes	0.43	1.30	0.92	0.29	0.17
Lentisphaerae	0.55^a^^A^	0.44^ab^^A^	0.11^b^^B^	0.14	0.03
Cyanobacteria	0.46^a^	0.25^ab^	0.13^b^	0.09	0.05
Tenericutes	0.48	0.64	0.40	0.17	0.79
Verrucomicrobia	0.27	0.25	0.58	0.25	0.96
Actinobacteria	0.14	0.14	0.53	0.24	0.51

a,b *Treatments that do not share a letter had significantly different results by Tukey's honestly significant difference (HSD) test at a P < 0.05, corrected for multiple comparisons*.

A,B *Treatments that do not share a letter had significantly different results by Tukey's honestly significant difference (HSD) test at a P < 0.10, corrected for multiple comparisons*.

Bacteroidetes and Tenericutes were less prevalent in rumen liquid digesta during the GBSC treatment compared to the APSC and control treatments. Compared to the control treatment, the GBSC treatment tended to decrease the relative abundance of Cyanobacteria. The abundance of Cyanobacteria differed between the GBSC and APSC treatments. The APSC treatment also tended to lower the abundance of SR1, but this reduction was greater during the GBSC treatment. In the cecum, only the GBSC treatment reduced the abundance of Lentisphaerae. In addition, the effect of the two SARA challenges on Verrucomicrobia was in the opposite direction with a slightly higher abundance in APSC. In the feces, the APSC only tended to reduce the abundance of Lentisphaerae and did not affect the abundance of Cyanobacteria, and, the GBSC treatment had lower Lentisphaerae and Cyanobacteria compared to the Control treatment.

#### Genus level

In rumen fluid samples, 71,029 sequences passed the quality check and were used for downstream bioinformatics analysis. A total of 36,178 sequences were classified into 60 genera. The APSC treatment did not affect the abundances of these genera. In contrast, the GBSC treatment increased the abundance of *Sharpea* and tended to increase those of *Ruminococcus, Megasphaera*, and *Shuttleworthia*, while it decreased those of *CF231*and *BF31* (Supplementary Table 2). In cecal digesta, 27,100 sequences of 94,252 sequences were classified into 106 genera, of these, only that of *Sharpea* was increased by the GBSC treatment (Supplementary Table 2). In the feces, 18,903 sequences of 104,277 sequences were classified into 73 genera. The GBSC treatment tended to increase the abundances the *CF231* and *YRC22* in feces and tended to decrease those of *Paludibacter* and *Epulopiscium* (Supplementary Table 2). The APSC treatment did not affect the abundances of any of the identified genera on the feces.

### Classical species quantified by quantitative PCR

**Figure 5:**
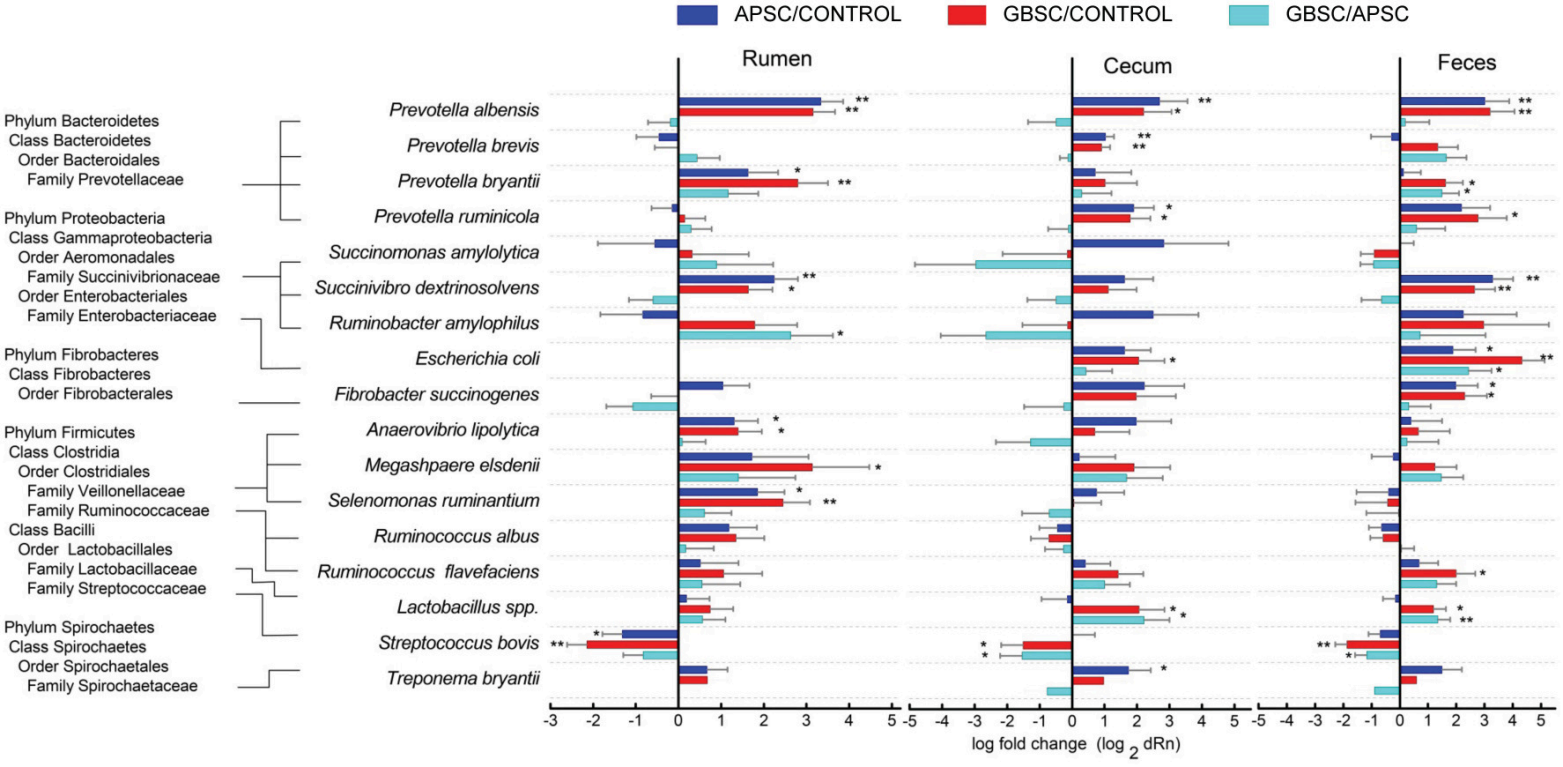
**Changes (log 2) in the abundances of 16 classical bacterial species and a group of Lactobacillus spp. during a grain-based SARA challenge (GBSC) and an alfalfa-pellet SARA challenge (APSC) in rumen liquid, cecal digesta, and feces determined by qPCR**. Symbols “^*^” and “^**^” indicate significance levels at *P* < 0.05 and *P* < 0.01, respectively.

The results of the qPCR of 16 bacterial species and a group of *Lactobacillus* spp. in rumen liquid, cecal digesta, and feces are given in Figure 5. In the rumen, both the GBSC and APSC treatments increased *Prevotella albensis, Prevotella bryantii, Succinivibrio dextrinosolvens, Anaerovibrio lipolytica, Selenomonas ruminantium*, but reduced *Streptococcus bovis*. Only the GBSC increased *Megasphaera elsdenii*. In the cecum, both the GBSC and APSC challenges increased *P. albensis, Prevotella brevis, Prevotella ruminicola, and Lactobacillus* spp., and decreased *S. bovis*. However, only the GBSC treatment increased *Escherichia coli*, and only the APSC treatment increased *Treponema bryantii*. In the feces, *P. albensis, S. dextrinosolvens, Fibrobacter succinogenes* and *Lactobacillus* spp. were increased and *S. bovis* was decreased by both the GBSC and APSC treatments. Only the GBSC treatment increased *P. ruminicola*, and *Ruminococcus flavefaciens*. In addition, both the GBSC and APSC treatments increased the abundance of *E. coli*, although the magnitude of the increase caused by GBSC was greater than that by APSC.

## Discussion

### Changes of the gut environment due to the APSC and GBSC challenges

Among the factors affecting bacteria in the gut environment, the most significant ones arguably are the capacity to utilize available nutrients and high growth rates to avoid washout and appease a reaction-ready immune system (Ley et al., 2006). In dairy cows, experimentally-induced SARA, either by feeding high-grain diets or by feeding pellets of ground forage, adversely impacts the environmental conditions in the rumen and the hindgut that can affect their bacteria (Plaizier et al., 2008, 2012; Khafipour et al., 2009a,b,c). The pH and concentrations of VFA and free LPS obtained in this experiment were reported in the companion paper from Li S. et al. (2012). In brief, the GBSC increased the duration of the rumen pH below 5.6 from 56.4 to 298.8 min/d, and free LPS in rumen fluid from 10,405 to 168,391 EU/ml. In addition, this challenge increased the concentration of free LPS in cecal digesta from 12,832 to 93,154 EU/ml, and the starch content of this digesta from 2.8 to 7.4% of DM. The GBSC also reduced the pH of cecal digesta from 7.07 to 6.79. The APSC caused reductions of the pH of rumen fluid, cecal digesta, and feces that were similar to those caused by the GBSC. In contrast to the GBSC, the APSC did not affect the free LPS and starch contents of cecal digesta, and only increased the free LPS content of rumen fluid to 12.832 to 30.715 EU/ml.

The above shows that in terms of the decrease in pH of digesta, both SARA challenges did not differ substantially (Li S. et al., 2012). The main differences between these challenges as reported in the companion manuscript of Li S. et al. (2012) were in starch and LPS contents of digesta in the foregut, hindgut and feces that were higher during the GBSC, and in agreement with the increase in starch feeding during this SARA challenge (Plaizier et al., 2008, 2012). These differences between the two SARA challenges suggests that the bacterial communities in the digestive tract could differ between these challenges, with larger populations of starch fermenting and LPS-shedding bacteria (gram-negatives) during the GBSC. The size of rumen pH depressions obtained by both challenges suggests that the induced acidosis was subacute and that no lactic acidosis and accumulation of lactate in rumen digesta occurred.

### Effects of SARA challenges on bacterial alpha- and beta-diversities

Both SARA challenges reduced the richness, diversity, and evenness of bacteria in the rumen, with higher magnitudes observed during GBSC. In addition, only the GBSC challenge reduced bacterial richness and diversity in the feces. In agreement, Fernando et al. (2010), Petri et al. (2013), and Mao et al. (2013) also reported that increasing the grain and starch contents of diets lowered the bacterial richness and diversity in the rumen of cattle. In these studies, these effects of high-grain feeding were more pronounced than in our study, which may be resulting from the higher increases in grain feeding in those studies.

It has been suggested that the bacterial communities that are high in species-richness and evenness use resources more efficiently, as species differ in their functionality and specialization to use fractions of the limiting substrate resources in the digestive tract (Levine and D'Antonio, 1999). Hence, the reduction in species richness and diversity during the SARA challenges, and especially during the GBSC, suggest that the rumen bacteria were transformed into less functional and desirable state.

Based on the UniFrac distances, the bacterial communities of the control and GBSC treatments clustered separately in rumen fluid, cecal digesta and feces, indicating significant impacts of high-grain feeding on gastrointestinal bacteria of dairy cows. These results agree with many earlier studies (Khafipour et al., 2009c; Fernando et al., 2010; Mao et al., 2013). The higher bacterial distance between the GBSC and control clusters than that between the control and the APSC clusters indicates that the magnitude of impacts does not solely depend on the reductions in the pH of digesta that these challenges cause, as these pH reductions did not differ between GBSC and APSC. Bacterial clustering based on the unweighted UniFrac distance provided a clearer separation than that based on the weighted UniFrac distance. Hence, the separation was reduced when the presence and abundance, rather than only the presence of bacteria were considered (Lozupone et al., 2012), which suggests that less abundant bacterial taxa are more affected by the SARA challenges than the more abundant ones.

### Changes of bacteria at different taxonomic levels

Similar to other mammals, the bacteria of rumen fluid, cecal digesta and feces in the dairy cows of our study were dominated by members of two bacterial phyla, i.e., Bacteroidetes and Firmicutes (Ley et al., 2008). The GBSC reduced the relative abundance of Bacteroidetes and, thereby, increased the Firmicutes to Bacteroidetes ratio in the rumen. However, the abundance of Bacteroidetes was not affected by the APSC. Mao et al. (2013) and Khafipour et al. (2009c) also studied the effect of a grain-based SARA challenge on the abundance of Bacteroidetes. The reductions in this abundance reported by Mao et al. (2013) and that observed by Khafipour et al. (2009c) in cows with severe SARA were greater than that found in our study. However, Khafipour et al. (2009c) reported a smaller reduction in cows with mild SARA. It still needs to be determined whether an increase in the Firmicutes to Bacteroidetes ratio, such as that observed in our study, is unfavorable for the functionality of the bacteria in the digestive tract of cattle. White et al. (2014) concluded that Firmicutes differ from Bacteroidetes in how they degrade plant biomass in the rumen, as Firmicutes degrade cell surfaces and the degradation of Bacteroidetes is mainly periplasmic and intracellular. El Kaoutari et al. (2013) concluded that, on average, Firmicutes encoded fewer glycan-cleaving enzymes than Bacteroidetes. This functional difference between these two phyla may explain and support the consideration that an increase in the Firmicutes to Bacteroidetes ratio in the rumen is undesirable.

In agreement with earlier studies (Petri et al., 2012, 2013; Mao et al., 2013), *Prevotella* was the most abundant genus of Bacteroidetes and *Ruminococcus* was the most abundant genus of Firmicutes in the rumen. Our study shows that the abundances of most bacterial genera that were classified in our study in the rumen, cecum, and feces were not affected by the SARA challenges, which agrees with the findings of Petri et al. (2012, 2013) and Mao et al. (2013).

The effects of the SARA challenges at the individual species level were determined by qPCR, as 16S rRNA gene sequencing based bacterial community profiling do not have sufficient resolution to determine treatments effects at the species level (McCann et al., 2014). The populations of amylolytic bacteria were expected to increase during the GBSC treatment, as this treatment increased availability of substrates for these bacteria in digesta in the rumen and the large intestine. In agreement, the GBSC increased the populations of *P. albensis, P. bryantii and S. ruminantium* in rumen liquid digesta, the population of *P. ruminicola* in cecal digesta, and that of *P. albensis* in feces. In contrast, the GBSC reduced the population of amylolytic *S. bovis* in the rumen, cecum, and feces. Despite not increasing the starch content of the diet, the APSC increases the populations of several amylolytic bacteria in the rumen, cecum and feces. Hence, changes in the availability of starch cannot only explain changes in populations of these bacteria. In order to explain the effects of APSC on these bacteria, effects of this treatment on rumen metabolomics and competition among various bacteria for substrates may be required.

Next to increasing *S. ruminantium*, GBSC increased, *S. dextrinosolvens*, and *A. lipolytica* in the rumen. A higher availability of pectin, dextrins, and sugars in the rumen resulting from the increase GBSC would be the reason for increased *S. dextrinosolvens* (Russell and Rychlik, 2001). *A. lipolytica* utilizes sugars, and the increase in their abundance during GBSC may, therefore, be explained by increased availabilities of these substrates.

A reduction in the dietary fiber content and in the rumen pH reduce the relative abundances of cellulolytic bacteria in the rumen (Shi and Weimer, 1992). Despite of this, the GBSC had no effects on the populations of cellulolytic *F. succinogenes, R. albus*, and *R. flavefaciens*. This finding may be due to a potential limitation in our study in which only rumen liquid digesta was analyzed. Cellulolytic bacteria are more associated with the solid than with the liquid digesta fraction (Petri et al., 2012, 2013). Hence, the impact of a SARA challenge on cellulolytic bacteria may not be evident when only liquid digesta is analyzed. A surprising finding was that both SARA challenges increased cellulolytic *F. succinogenes* in feces. However, as the rumen pH depressions during both SARA challenges may have reduced digestion of cellulose in the rumen, they may have increased the amount of cellulose, and, thereby, the substrates for cellulolytic bacteria in the hindgut.

As *S. bovis* is a starch utilizer and pH tolerant bacterium (Russell and Hino, 1985), the grain-based SARA challenges would be expected to increase its abundance. However, both the GBSC and the APSC reduced its abundance in rumen fluid, and GBSC reduced its abundance in cecal digesta and feces. Tajima et al. (2001) and Petri et al. (2013) also reported similar findings in the rumen during excessive grain feeding to cattle. An explanation for this may be that the abundance of this bacterium only increases during severe and lactic rumen acidosis (Khafipour et al., 2009c), and that the pH and lactic acid concentrations of rumen fluid in our study did not indicate that lactic acidosis was induced. The increase in the abundance of *M. elsdenii* in the rumen fluid during the GBSC also confirms the increased production of lactate and sugars and that the induced SARA was not severe (Russell and Rychlik, 2001; Khafipour et al., 2009c). Increases in the population of *E. coli* due to high-grain feeding, such as that seen in our study, have been described earlier (Diez-Gonzalez et al., 1998; Khafipour et al., 2011). These authors did not only observe an increase in the population of this species, but also in the population of more acid-resistant and virulent *E. coli* strains.

### Relationships between bacterial populations and LPS

The companion study by Li S. et al. (2012) reported that the GBSC increased the concentration of LPS in rumen fluid by 16.0-fold. In our study, the GBSC decreased the relative abundance of Bacteroidetes in the rumen by 16% (from 48.9% to 41.9% of the community) but did not change the abundance of Proteobacteria and Fibrobacteres. In the cecum and feces, the abundances of Bacteroidetes and Proteobacteria were not affected by the GBSC, whereas the LPS concentration in cecal digesta and feces increased during this treatment. Treatments effects on the abundances of several bacterial species were observed, but as only a selection of gram-negative species were monitored by PCR, changes in the abundances of these species may not be the sole cause of the observed changes in the LPS content of digesta. The limulus amoebocyte lysate assay used in the parallel study (Li S. et al., 2012) is a bioassay that is based not on the concentration of LPS, but on the bioactivity of this LPS, which varies among bacterial species (Plaizier et al., 2012). This assay also does not indicate the source of the LPS (Plaizier et al., 2012). This information is important, as the toxicity of LPS varies among gram-negative bacterial species (Plaizier et al., 2012). Hence, changes in the populations of these bacteria do not have to cause changes in the concentration of LPS as they were reported by Li S. et al. (2012).

Functional changes in bacteria, such as the changes in the growth and lysis rates of several species of gram-negative bacteria, are likely to be responsive for the effects of both SARA challenges on LPS concentrations in digesta (Plaizier et al., 2012), but the resolution of the sequencing used in our study was not sufficient to assess these the effects of treatments on bacterial species (McCann et al., 2014). This shows that in order to assess the effects of dietary changes on the functionality of bacteria, techniques that can determine changes in the metagenome, such as whole genome shotgun sequencing, need to be used. The relationship between concentration of free LPS and the populations of LPS containing bacteria in digesta, therefore, remains not fully understood.

## Conclusions

The APSC and the GBSC both reduced the bacterial richness and diversity in rumen fluid, but the GBSC had a larger effect. The bacterial community of GBSC also clustered differently from control feeding in rumen fluid, cecal digesta and feces. The bacterial community of APSC also clustered differently from control feeding in rumen fluid and feces, but not in cecal digesta. Despite of this, only GBSC reduced bacterial richness and diversity in feces. The abundances of Bacteroidetes and Tenericutes in rumen fluid were decreased by GBSC, but not by APSC. Effects of the GBSC on the abundances of bacterial genera in the rumen, cecum and feces were also limited. The APSC did not affect any of these abundances. Both challenges increased the abundances of several bacteria that utilize non-structural carbohydrates and their metabolites in the rumen, cecum, and feces to a larger extent than the genera and phyla to which they belong, but both challenges decreased the abundance of *S. bovis*. Only GBSC increased the abundance of *M. elsdenii* in the rumen. Differences in the starch content of rumen and hindgut digesta between the GBSC and the APSC as reported in the companion manuscript from Li S. et al. (2012) may have contributed to the dissimilarities in the gut bacteria with regard to the differing SARA-induction challenges.

## Ethics statement

The study were approved by the University of Manitoba Animal Care Committee according to guidelines of the Canadian Council on Animal Care (1993).

## Author contributions

JP, EK, and SL conceived and designed the experiment. SL performed the experiment. EK and SL performed lab analyses. EK, SL, HT, and JP analyzed the data. All authors drafted the manuscript. All authors carefully read and approved the final version of the manuscript.

## Funding

The research was funded by the National Sciences and Engineering Research Council of Canada (NSERC), Dairy Farmers of Manitoba (DFM), and Agri-Food Research and Development Initiative (ARDI).

### Conflict of interest statement

The authors declare that the research was conducted in the absence of any commercial or financial relationships that could be construed as a potential conflict of interest.
