# Supplementary material for: Nutritional Models of Experimentally-Induced Subacute Ruminal Acidosis (SARA) Differ in Their Impact on Rumen and Hindgut Bacterial Communities in Dairy Cows
Source: Front Microbiol. 2017 Jan 25;7:2128. doi: 10.3389/fmicb.2016.02128 (PMC5265141; doi:10.3389/fmicb.2016.02128)
Supplement: Supplementary file 1 [file Table1.DOCX]

**Table S1.** PCR primers used in this study

| Target taxon /species tested | Primer set | Primer sequences | length | A_Temp | GC% | Amplicon size | Effici-ency | Source of primer |
| --- | --- | --- | --- | --- | --- | --- | --- | --- |
| Domain bacteria | 341-357F | CCTACGGGAGGCAGCAG | 17 | 55.2 | 70.6 | 189 | 2.03 | (Muyzer et al., 1993) |
|  | 518-534R | ATTACCGCGGCTGCTGG | 17 | 56.2 | 64.7 |  |  |  |
| Lactic Acid Bacteria | Ulac16S1F | AGCAGTAGGGAATCTTCCA | 19 | 51.5 | 47.4 | 345 | 1.9 | (Walter et al., 2001, Lan et al., 2004) |
|  | Ulac16S1R | ATTCCACCGCTACACATG | 18 | 51.1 | 50.0 |  |  |  |
| Streptococcus bovis | SBovis2F | ATTCTTAGAGATAGGGTTTCTCTT | 24 | 64.0 | 33.3 | 109 | 1.99 | (Fernando et al., 2010) |
|  | SBovis2R | ACCTTATGATGGCAACTAACAATA | 24 | 64.0 | 33.3 |  |  |  |
| Succinivibrio dextrinosolvens | SucDex1F | TAGGAGCTTGTGCGATAGTATGG | 23 | 57.4 | 47.8 | 174 | 1.92 | (Khafipour et al., 2009) |
|  | SucDex1R | CTCACTATGTCAAGGTCAGGTAAGG | 25 | 58.4 | 48.0 |  |  |  |
| Selenomonas ruminantium | SelRum1F | GGCGGGAAGGCAAGTCAGTC | 20 | 60.4 | 65.0 | 83 | 1.96 | (Khafipour et al., 2009) |
|  | SelRum1R | CCTCTCCTGCACTCAAGAAAGACAG | 25 | 61.1 | 52.0 |  |  |  |
| Megasphaera elsdenii | MegEls1F | GACCGAAACTGCGATGCTAGA | 21 | 57.7 | 52.4 | 129 | 1.85 | (Ozutsumi et al., 2006) |
|  | MegEls1R | CGCCTCAGCGTCAGTTGTC | 19 | 58.2 | 63.2 |  |  |  |
| Anaerovibrio lipolytica | AnaLip2F | TGGGTGTTAGAAATGGATTCTAGTG | 25 | 56.6 | 40.0 | 109 | 1.98 | (Khafipour et al., 2009) |
|  | AnaLip2R | GCACGTCATTCGGTATTAGCAT | 22 | 56.7 | 45.5 |  |  |  |
| Ruminobacter amylophilus | RumAmy2F | CTGGGGAGCTGCCTGAAT | 18 | 55.3 | 61.1 | 100 | 1.92 |  |
|  | RumAmy2R | CATCTGAATGCGACTGGTTG | 20 | 54.2 | 50.0 |  |  |  |
| Prevotella brevis | PreBre1F | GCTTGCTTTTGAAGATGGCGAC | 22 | 58.8 | 50.0 | 153 | 1.85 | (Khafipour et al., 2009) |
|  | PreBre1R | CATCCCTTAGCGATAAATCTTTGCT | 25 | 57.6 | 40.0 |  |  |  |
| Prevotella albensis | ProAlb4F | GCGCCACTGACGCTGAAG | 18 | 58.3 | 66.7 | 110 | 1.86 | (Khafipour et al., 2009) |
|  | ProAlb4R | CCCCAAATCCAAAAGGACTCAG | 22 | 56.6 | 50.0 |  |  |  |
| Treponema bryantii | TrpBry1F | GAGAAACGCTTTGTGGTGACTGT | 23 | 59.5 | 47.8 | 122 | 1.95 | (Khafipour et al., 2009) |
|  | TrpBry1R | CCTACATGCCCTTTACGCTCAAT | 23 | 58.7 | 47.8 |  |  |  |
| Fibrobacter succinogenes | FibSuc4F | GGAGCGTAGGCGGAGATTCA | 20 | 58.7 | 60.0 | 97 | 1.99 | (Khafipour et al., 2009) |
|  | FibSuc4R | GCCTGCCCCTGAACTATCCA | 20 | 58.5 | 60.0 |  |  |  |
| Ruminococcus albus | RumAlb1F | CCCTAAAAGCAGTCTTAGTTCG | 22 | 54.3 | 45.5 | 176 | 1.97 | (Wang et al., 1997) |
|  | RumAlb1R | CCTCCTTGCGGTTAGAACA | 19 | 53.8 | 52.6 |  |  |  |
| Ruminococcus flavefaciens | RumFla1F | CGAACGGAGATAATTTGAGTTTACTTAGG | 29 | 57.5 | 34.5 | 132 | 1.99 | (Denman and McSweeney, 2006) |
|  | RumFla1R | CGGTCTCTGTATGTTATGAGGTATTACC | 28 | 59.3 | 42.9 |  |  |  |

Denman, S. E. and C. S. McSweeney. 2006. Development of a real-time PCR assay for monitoring anaerobic fungal and cellulolytic bacterial populations within the rumen. FEMS Microbiol Ecol 58(3):572-582.

Khafipour, E., S. Li, J. C. Plaizier, and D. O. Krause. 2009. Rumen microbiome composition determined using two nutritional models of subacute ruminal acidosis. Appl. Environ. Microbiol. 75(22):7115-7124.

Lan, Y., S. Xun, S. Tamminga, B. A. Williams, M. W. Verstegen, and G. Erdi. 2004. Real-time PCR detection of lactic acid bacteria in cecal contents of eimeria tenella-lnfected broilers fed soybean oligosaccharides and soluble soybean polysaccharides. Poult Sci 83(10):1696-1702.

Muyzer, G., E. C. de Waal, and A. G. Uitterlinden. 1993. Profiling of complex microbial populations by denaturing gradient gel electrophoresis analysis of polymerase chain reaction-amplified genes coding for 16S rRNA. Appl. Environ. Microbiol. 59(3):695-700.

Ozutsumi, Y., K. Tajima, A. Takenaka, and H. Itabashi. 2006. Real-time PCR detection of the effects of protozoa on rumen bacteria in cattle. Current microbiology 52(2):158-162.

Walter, J., C. Hertel, G. W. Tannock, C. M. Lis, K. Munro, and W. P. Hammes. 2001. Detection of Lactobacillus, Pediococcus, Leuconostoc, and Weissella species in human feces by using group-specific PCR primers and denaturing gradient gel electrophoresis. Applied and environmental microbiology 67(6):2578-2585.

Wang, R. F., W. W. Cao, and C. E. Cerniglia. 1997. PCR detection of Ruminococcus spp. in human and animal faecal samples. Mol Cell Probes 11(4):259-265.
