# Supplementary material for: Nutritional Models of Experimentally-Induced Subacute Ruminal Acidosis (SARA) Differ in Their Impact on Rumen and Hindgut Bacterial Communities in Dairy Cows
Source: Front Microbiol. 2017 Jan 25;7:2128. doi: 10.3389/fmicb.2016.02128 (PMC5265141; doi:10.3389/fmicb.2016.02128)
Supplement: Supplementary file 2 [file Table2.DOCX]

|  | **Supplementary Table 2.** Abundant genera (above 1% of population) in rumen fluid, and feces of dairy cows whose abundances were affected by treatment. Treatments included a control diet (Control), an alfalfa pellet SARA challenge (APSC) or a grain based SARA challenge (GBSC). The bacteria genera were classified using V1–V3 sequences of 16S rRNA gene in the pyrosequencing library. | | | | | | | | | |
| --- | --- | --- | --- | --- | --- | --- | --- | --- | --- | --- |
| Taxa present in each compartment | | |  | Percentage of sequences in: | | | SEM | P-values |  |  |
| Phylum | | Family, Genus |  | Control | APSC | GBSC |  |  |  |  |
| *Rumen* | |  |  |  |  |  |  |  |  |  |
| Bacteroidetes | | [Paraprevotellaceae], *CF231* |  | 1.6^abA^ | 1.8^aA^ | 0.6^bB^ | 0.48 | 0.04 |  |  |
| Firmicutes | | Erysipelotrichaceae, *Sharpea* |  | 0.1^bB^ | 0.6^bAB^ | 6.2^aA^ | 2.58 | 0.03 |  |  |
| Firmicutes | | Ruminococcaceae, *Ruminococcus* |  | 7.8^B^ | 6.4^B^ | 21.9^A^ | 9.57 | 0.08 |  |  |
| Bacteroidetes | | Bacteroidaceae, *BF311* |  | 0.51A | 0.16B | 0.17AB | 0.22 | 0.07 |  |  |
| Firmicutes | | Lachnospiraceae, *Shuttleworthia* |  | 0.02^bB^ | 0.18^abB^ | 0.64^aA^ | 0.25 | 0.07 |  |  |
| Firmicutes | | Veillonellaceae, *Megasphaera* |  | 0.004^B^ | 0.000^B^ | 0.053^A^ | 0.05 | 0.08 |  |  |
| *Cecum* | |  |  |  |  |  |  |  |  |  |
| Firmicutes | | Erysipelotrichaceae,Sharpea |  | 0.007^b^ | 0.007^b^ | 0.198^a^ | 0.08 | 0.04 |  |  |
| *Feces* | |  |  |  |  |  |  |  |  |  |
| Bacteroidetes | | [Paraprevotellaceae],*CF231,* |  | 3.0^B^ | 2.2^B^ | 4.5^A^ | 1.3 | 0.08 |  |  |
| Bacteroidetes | | [Paraprevotellaceae],*YRC22,* |  | 0.27^B^ | 0.14^B^ | 0.39^A^ | 0.19 | 0.09 |  |  |
| Bacteroidetes | | Porphyromonadaceae,*Paludibacter,* |  | 0.25^A^ | 0.24^AB^ | 0.09^B^ | 0.10 | 0.08 |  |  |
| Firmicutes | | Lachnospiraceae,*Epulopiscium,* |  | 0.51^A^ | 0.49^A^ | 0.10^B^ | 0.21 | 0.07 |  |  |
|  | ^a,b^ Treatments that do not share a letter had significantly different results by Tukey's honestly significant difference (HSD) test at a P value of <0.05, corrected for multiple comparisons.  ^A,B^ Treatments that do not share a letter had significantly different results by Tukey's honestly significant difference (HSD) test at a P value of <0.10, corrected for multiple comparisons. | | | | | | | | | |
