# Supplementary figures and images for: Nutritional Models of Experimentally-Induced Subacute Ruminal Acidosis (SARA) Differ in Their Impact on Rumen and Hindgut Bacterial Communities in Dairy Cows
Source: Front Microbiol. 2017 Jan 25;7:2128. doi: 10.3389/fmicb.2016.02128 (PMC5265141; doi:10.3389/fmicb.2016.02128)

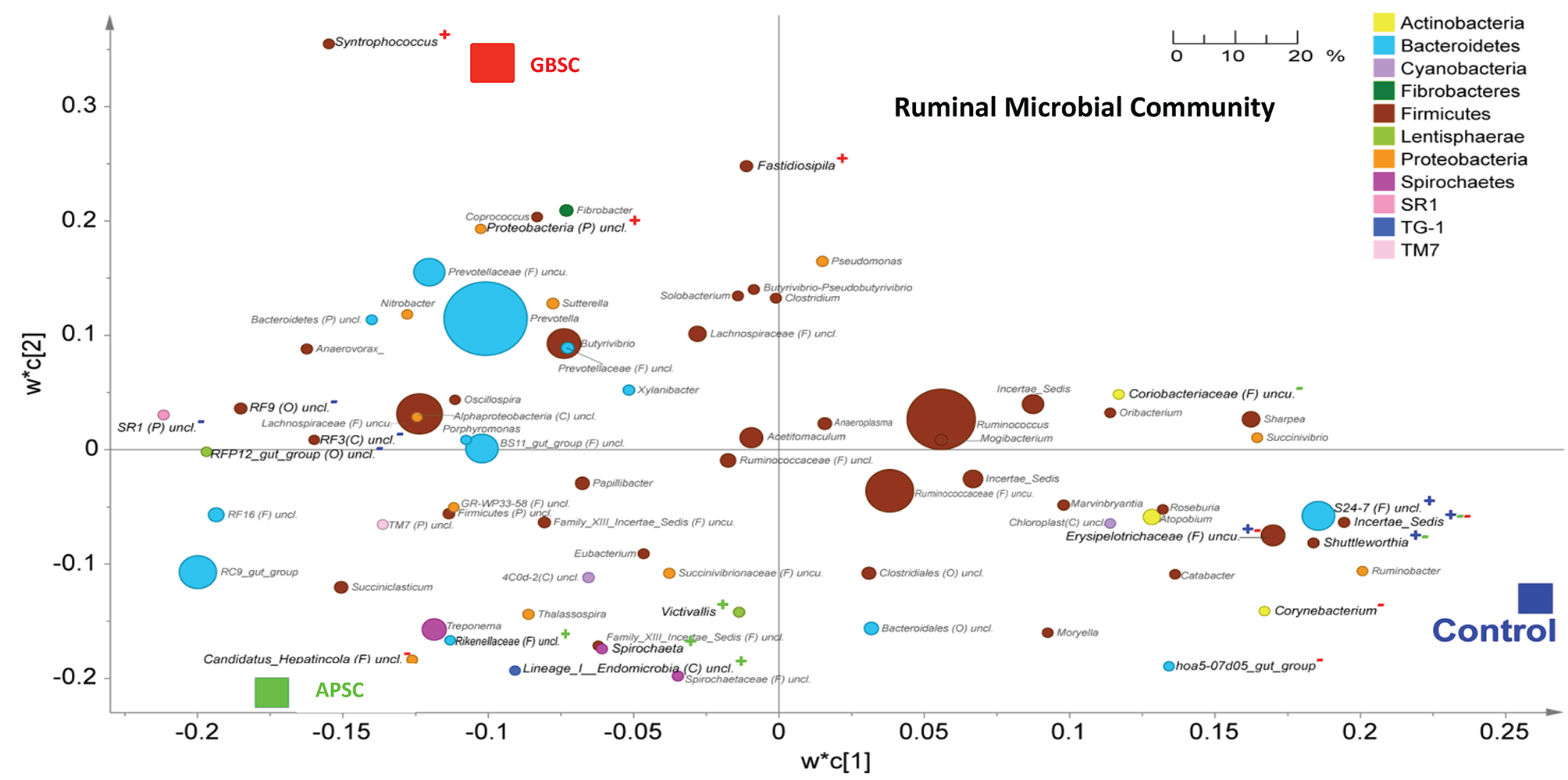

Supplement: Supplementary file 4 [file Image1.tiff]

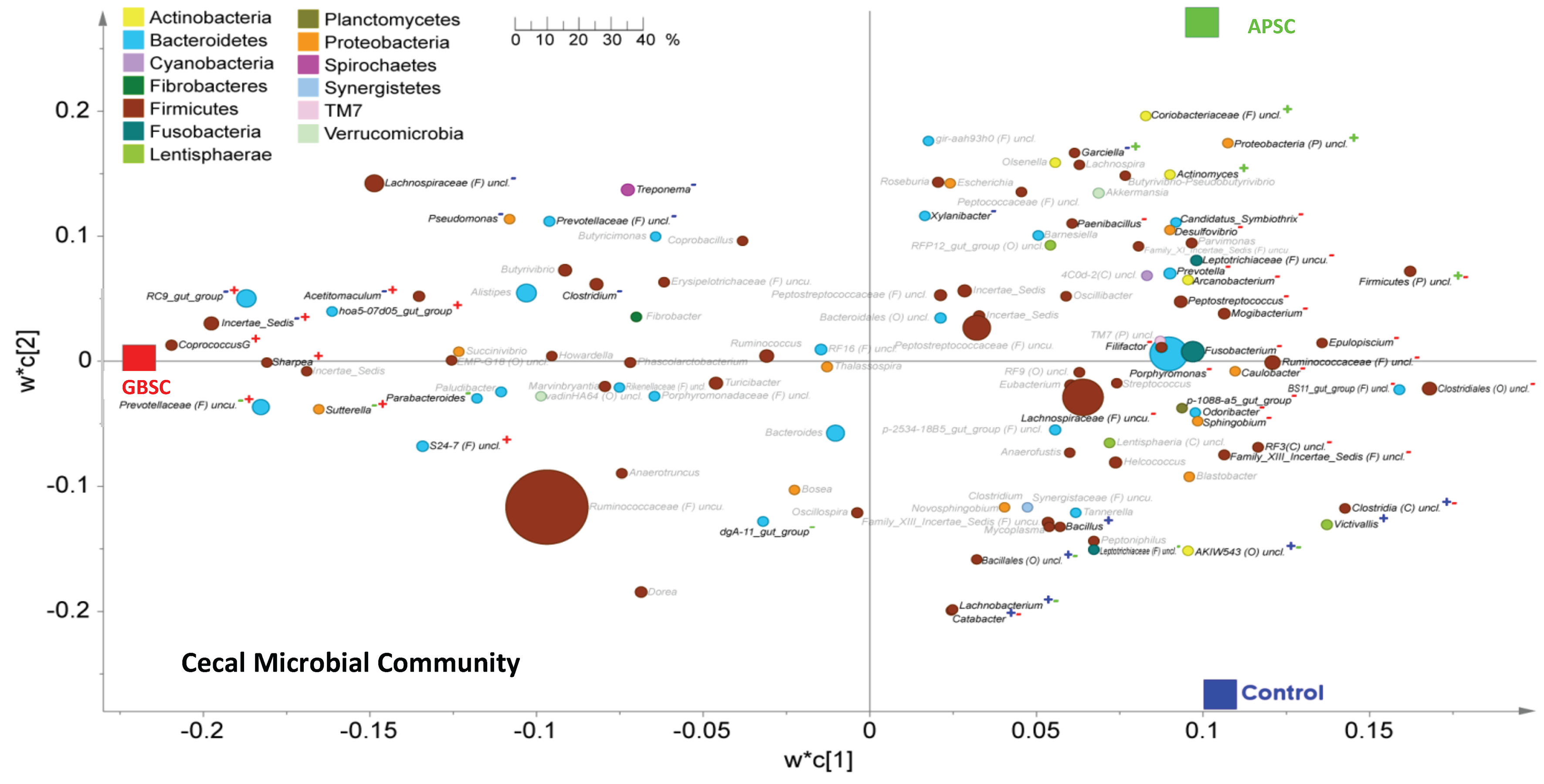

Supplement: Supplementary file 5 [file Image2.tiff]

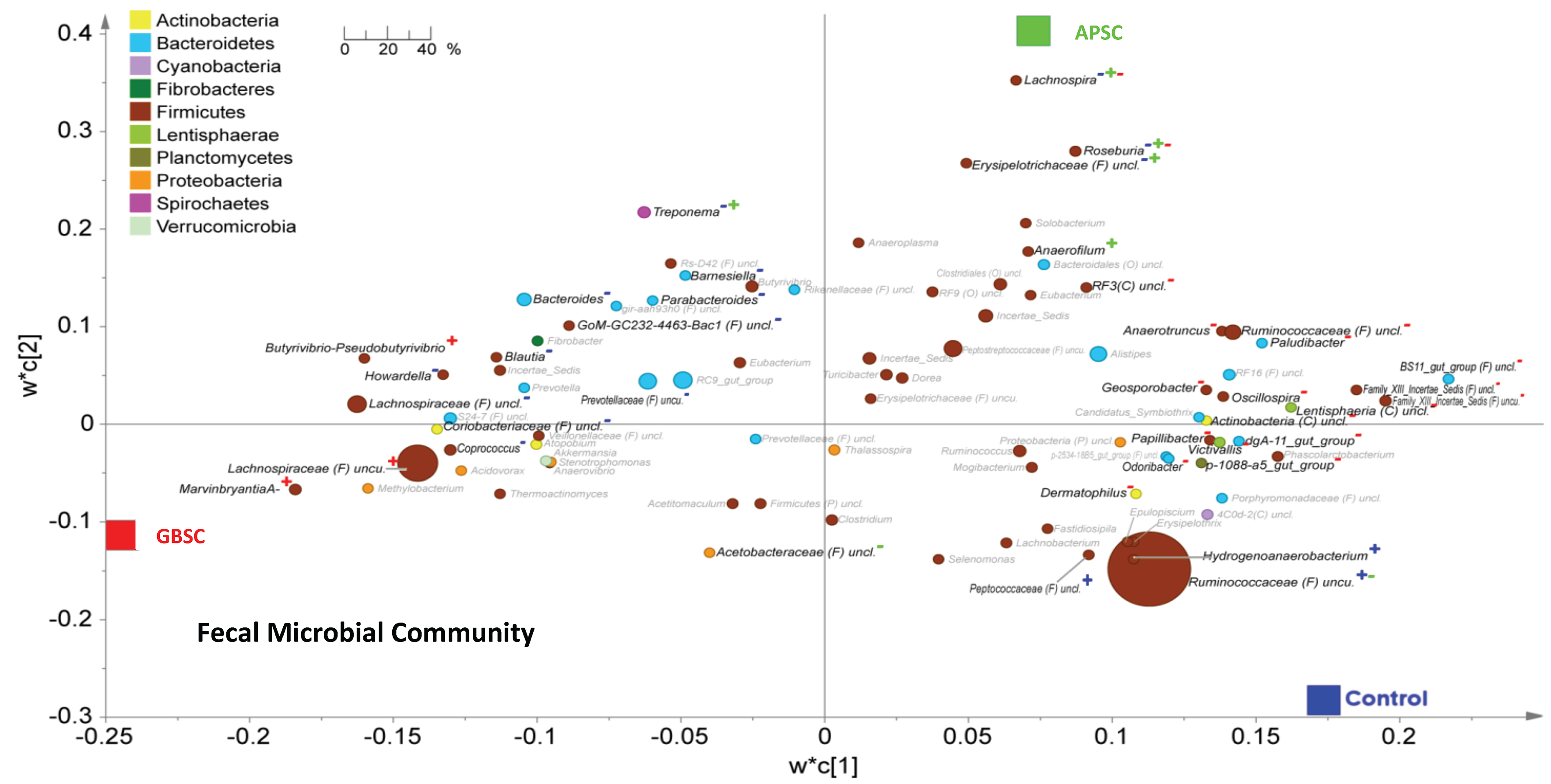

Supplement: Supplementary file 6 [file Image3.tiff]
